# Cysteine-reactive mitigators of small vessel disease-related NOTCH3 mutants

**DOI:** 10.1038/s41598-026-45103-1

**Published:** 2026-03-20

**Authors:** Naw May Pearl Cartee, Xiaojie Zhang, Soo Jung Lee, Michael M. Wang

**Affiliations:** 1 Department of Neurology, University of Michigan, 1137 Catherine St., Box 5622, Ann Arbor, MI 48109-5622 USA; 2 Departments of Molecular and Integrative Physiology, 7725 Medical Science Building II, University of Michigan, 1137 Catherine St., Box 5622, Ann Arbor, MI 48109-5622 USA; 3 Department of Veterans Affairs, Neurology Service, VA Ann Arbor Healthcare System, Ann Arbor, MI 48105 USA

**Keywords:** CADASIL, NOTCH3, Disulfiram, Auranofin, Split luciferase, Conformational change, Cysteines, Disulfide bonds, Biochemistry, Drug discovery, Molecular biology, Neuroscience

## Abstract

Pathogenic alterations in NOTCH3 cause CADASIL, an accelerated and currently untreatable form of cerebrovascular disease. CADASIL mutant NOTCH3, which frequently harbors abnormalities in EGF repeat cysteine number, adopt disulfide dependent abnormal conformations. To seek potential strategies to mitigate the impact of CADASIL mutations on NOTCH3, we investigated whether cysteine-targeting compounds may affect pathological NOTCH3. LSL-NOTCH3, a split luciferase assay that discriminates between benign and pathogenic NOTCH3 conformations, was used to quantify the capacity of 21 small molecule compounds to restore NOTCH3 reporter activity. We assayed the activity of each of the small molecules on 16 different pathogenic mutants distributed over three different regions of NOTCH3. Ten of 21 compounds had statistically significant effects on at least one mutant NOTCH3. Five of 21 compounds had mitigating effects on a majority of mutant NOTCH3 in three regions of the protein, with disulfiram and auranofin targeting the most favorable range of mutants. These findings support the concept that cysteine-targeting can potentially mitigate the effects of a broad range of NOTCH3 pathogenic mutants and provide an impetus to investigate whether cysteine-targeting strategies affect disease-relevant phenotypes in EGF repeat-related human disorders.

**Supplementary Information:**

The online version contains supplementary material available at 10.1038/s41598-026-45103-1.

## Introduction

Missense variants in *NOTCH3* are the most common and significant causes of inherited small vessel disease. In particular, cysteine alterations in NOTCH3 cause CADASIL, an accelerated form of cerebrovascular disease that features progressive cognitive impairment and early onset stroke^1,2^. Non-canonical, non-cysteine altering missense mutations in NOTCH3 also contribute to a small fraction of CADASIL^3–6^. In both cysteine-altered and non-canonical CADASIL, pathological features of proteinopathy include accumulation of mutant NOTCH3 protein and of granular osmiophilic material (GOM) in the extracellular matrix of blood vessel cells^7,8^.

Because almost all CADASIL mutations result in either a loss or a gain of a cysteine residue, it has been proposed that abnormal disulfide bonding contributes to the pathogenic process. Specifically, disulfide-dependent aberrant secondary structure in CADASIL NOTCH3 protein has been posited to alter protein clearance and subsequently disturb physiology of brain vessels^9^. Whether non-cysteine CADASIL mutants also harbor disulfide abnormalities is not clear.

In prior work, we have found that CADASIL mutant NOTCH3 folds into conformations that migrate abnormally slowly in polyacrylamide gels^10^. These conformations require disulfide bridges, indicating that aberrant cysteine bonds drive protein folding abnormalities that are linked to disease. Notably, non-canonical, non-cysteine altering mutants also migrate abnormally in gels only in non-reducing conditions, suggesting that abnormal disulfide bridges may be a common feature of all pathogenic NOTCH3 mutants.

In subsequent work, we developed the light chain split luciferase-NOTCH3 assay (LSL-NOTCH3) that quantitatively discriminates between benign and pathogenic NOTCH3 variants using multiple parameters^11^. Parameter 1 of the LSL-NOTCH3 assay provides a composite assessment of protein production, secretion, and folding of a NOTCH3 variant. Parameter 2 of the LSL-NOTCH3 assay provides an assessment of the fraction of NOTCH3 that is properly disulfide linked. By comparing these two parameters for wildtype NOTCH3 versus any variant of NOTCH3, this assay provides an opportunity to identify amino acid residues that affect NOTCH3 production and/or NOTCH3 folding.

Analysis of double mutants of NOTCH3 indicated that the impact of single cysteine mutation pathological conformations could be suppressed by eliminating specific cysteines in CADASIL mutant proteins, which is consistent with the concept that free thiol groups participate in aberrant NOTCH3 conformations^10,11^. In addition, the thiol labeling reagent iodoacetamide (IAM) was noted to reduce the impact of CADASIL mutations in LSL-NOTCH3 assays^11^. As such, we reasoned that a potential strategy to reduce the pathological impact of NOTCH3 mutations could be to apply small molecules that covalently target unreacted thiol groups.

To search for potential cysteine-targeting compounds that affect pathological NOTCH3, we leveraged the LSL-NOTCH3 assay to screen a collection of cysteine-reactive small molecules. Small cysteine modifying chemicals were evaluated for their ability to mitigate abnormal mutant LSL-NOTCH3 production or folding. Furthermore, each candidate was evaluated against a panel of pathogenic LSL-NOTCH3 variants to determine the targeting range of cysteine modifying chemicals across multiple EGF domains of NOTCH3.

## Results

### Small molecule screening

The 21 compounds selected for study were molecules with capacity to react with protein thiols. We included two well-established cysteine alkylation agents that are expected to have broad target range, iodoacetamide (IAM) and N-ethylmaleimide (NEM). Other reagents included FDA-approved or investigational drugs with ability to alkylate cysteines; we hypothesized that these compounds would act on a more restricted range of NOTCH3 variants. The largest groups of compounds were oncological medications (that bind pro-proliferative proteins via cysteines) and proton pump inhibitors that covalently modify gastric H + /K + ATPase (via a sulfenic acid group). The compounds and their chemical features are displayed in Table 1.

**Table 1:** Twenty one cysteine-reactive candidates tested for ability to mitigate NOTCH3 conformational alterations. Compounds are listed in order of chemical complexity, which was obtained from https://pubchem.ncbi.nlm.nih.gov/compound. Lipophilicity data was calculated from https://www.swissadme.ch. Electrophilicity data was determined from https://www.esnuel.org. H = exceeds calculation limits. The last two columns show the number of pathogenic variant reporters significantly increased in activity out of the 16 tested. Of FDA approved drugs, disulfiram and auranofin exhibited the most widespread effects.

Chemical name	Chemical reactivity	FDA approval	Complexity	Lipophilicity	Electrophilicity	Parameter 1 mitigation	Parameter 2 mitigation
2-Iodoacetamide (IAM)	Nucelophilic substitution (SN2) reaction	No	44.9	0.16	248.19	13	10
2-(butan-2-yldisulfanyl)-1H-imidazole (PX-12)	Nucelophilic substitution (SN2) reaction	No	111	2.14	167	10	9
N-acetylcysteine amide	Thiol disulfide exchange reaction	No	149	-0.44	125	0	0
N-ethylmaleimide (NEM)	Michael addition	No	165	0.48	253	10	7
Disulfiram (DSF)	Thiol disulfide exchange reaction	Yes	201	3.25	192	14	7
4-(2-Aminoethyl)benzenesulfonyl fluoride hydrochloride (AEBSF)	Nucelophilic substitution(SN2) reaction	No	239	1.62	568.51	0	0
Ebselen	Covalent bonding, forming a thioselenide linkage	No	275	1.75	185	3	11
Carmofur	Covalent adduct formation, carbamoylation	No	382	1.93	246	1	1
Rabeprazole	Sulfenamide intermediate, then Covalent disulfide bond	Yes	440	2.26	184	0	0
Tenatoprazole	Sulfenamide intermediate, then Covalent disulfide bond	No	455	2.05	250	0	0
Omeprazole	Sulfenamide intermediate, then Covalent disulfide bond	Yes	459	2.31	234	0	1
Lansoprazole	Sulfenamide intermediate, then Covalent disulfide bond	Yes	480	3.13	241	0	1
Pantoprazole	Sulfenamide intermediate, then Covalent disulfide bond	Yes	490	2.3	230	0	1
Auranofin	Ligand exchange reaction	Yes	532	1.12	H	12	5
Spebrutinib	Michael addition	No	561	3.41	H	3	5
Evobrutinib	Michael addition	No	595	3.48	H	0	0
Osimertinib	Michael addition	Yes	725	3.24	H	1	6
Ibrutinib	Michael addition	Yes	726	3.25	H	0	5
Zanubrutinib	Michael addition	Yes	728	3.17	H	0	1
Necrosulfonamide	Michael addition	No	760	1.53	H	7	3
Sotorasib	Michael addition	Yes	1030	4.1	H	0	1

### NOTCH3 variant evaluation platform

**Fig. 1:**
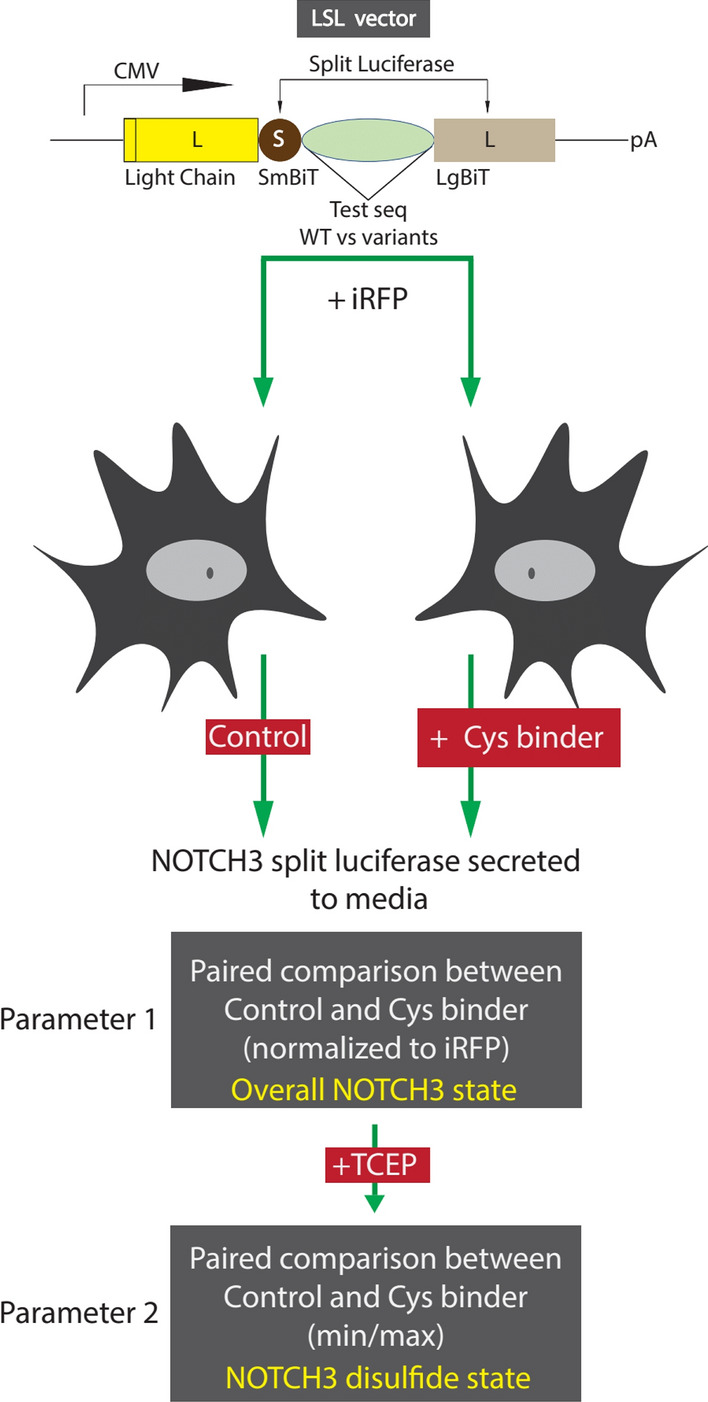
Experimental approach to evaluating potential mitigators of pathogenic NOTCH3 variants. To assess the potential beneficial effects of cysteine-binding small molecules on pathogenic variants of NOTCH3, we tested a series of candidates (Table 1) on a luciferase reporter (LSL-NOTCH3) that reflects normal folding of NOTCH3. In this system, pathogenic NOTCH3 mutants generate lower luciferase activity. We evaluated candidate agents for their ability to increase luciferase activity of pathogenic NOTCH3 reporters. (Top) Schematic of the LSL vector in which a CMV reporter drives expression of a recombinant protein composed of an antibody light chain (L in yellow), small BiT of nanoLuciferase (S), EGF repeats of NOTCH3 (WT vs mutant for EGF1-3, EGF4-6 or EGF31-33), and large BiT of nanoLuciferase (L in light brown). Cotransfection of iRFP is used to normalize transfection efficiency. Abbreviations include SmBiT (small bit of nanoluciferase), LgBiT (large bit of nanoluciferase), iRFP (infrared fluorescent protein), and TCEP (Tris (2-carboxyethyl) phosphine). Please see text for detailed description. Chemicals which increased mutant reporter Parameter 1 and/or Parameter 2 are considered mitigators of pathogenic NOTCH3 conformation.

As demonstrated in prior work, the LSL-NOTCH3 platform enables differentiation of benign variants and pathogenic variants of NOTCH3^11^. The platform (Fig. 1) employs human NOTCH3 variants (three EGF-like repeats at a time) that are cloned into the LSL backbone vector and then transfected into 293 cells along with an iRFP expression vector to normalize for transfection efficiency. High activity in the conditioned media is generated by WT and benign variants of NOTCH3; substantially lower activity is found after transfection with pathological NOTCH3 mutants. Impaired activity of mutants is a result of a combination of deficient expression levels, secretion efficiency, and/or conformational alterations that block luciferase activity reconstitution; total production of protein with retained luciferase activity is reflected by composite Parameter 1, the total luciferase secreted, normalized to iRFP levels. Conformational alterations that depend on disulfide bonding are reflected by Parameter 2, the ratio of unreduced luciferase activity normalized to total activity after unlocking aberrant disulfide bonds using the reductant TCEP.

Figure 1 diagrams how LSL-NOTCH3 was used to evaluate the degree to which candidate drugs attenuate the impact of pathological NOTCH3 mutants. LSL-NOTCH3 was first transfected into cells with iRFP. After an overnight incubation, candidate drugs in fresh media were overlayed onto cells. The expression and activity of the LSL-NOTCH3 constructs were determined 2 h after addition of fresh media (with DMSO or drug; Parameter 1). The ratio of expression with drug versus DMSO was calculated to measure the impact of drug. To determine the ability of the drug to block disulfide dependent pathological changes, Parameter 2 was determined for DMSO or drug-treated groups by adding TCEP to the luciferase assay and following the increase in activity over time.

### Attenuation of pathogenic properties of NOTCH3 by pharmacological agents

**Fig. 2:**
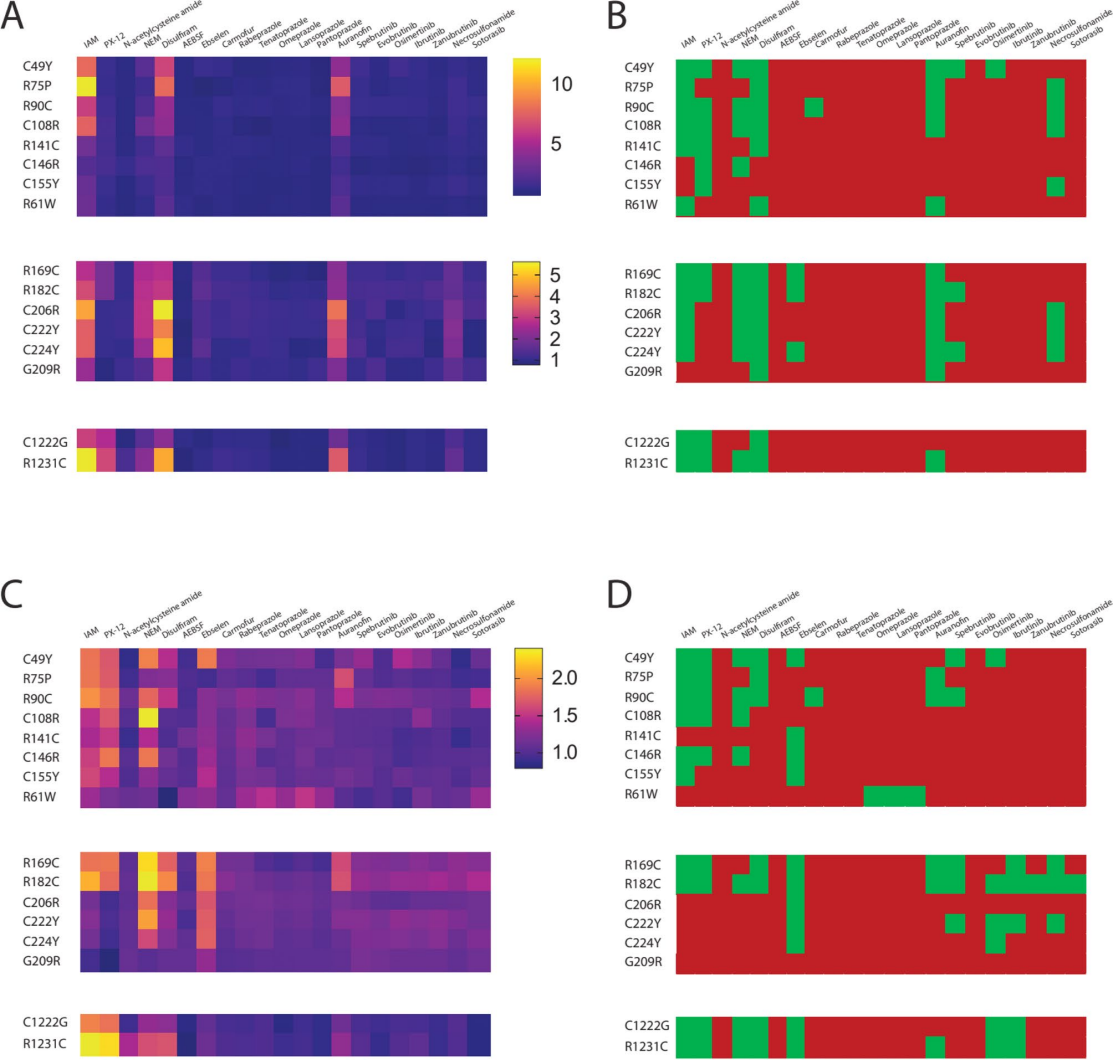
Pathogenic NOTCH3 conformational mitigation across mutants by a series of candidate small molecules. Using the workflow outlined in Fig. 1 and in methods, the reporter output of cells transfected with mutants shown on the vertical axis was determined after individual treatment with candidate mitigator drugs shown on the horizonal axis. Three different clusters of EGF repeats of NOTCH3 were examined: 1–3, 4–6, and 31–33. The ratio of Parameter 1 (total secreted luciferase/iRFP) for drug vs control conditions was then normalized to the respective WT ratio and displayed in the heat map in (**A**). All experiments were performed at least three times for each mutant and drug combination (biological replicates). Statistically significant increases in drug stimulated Parameter 1 over control are shown in (**B**). (**C**) A similar analysis of relative increases of Parameter 2 with treatments shown. (**D**) Statistically significant increases in Parameter 2 over control after drug treatments. In (B) and (D), green indicates p < 0.05.

We evaluated the effect of 21 cysteine acting agents on CADASIL mutants of NOTCH3 in EGF1-3, EGF4-6, and EGF31-33 (Fig. 2), regions that correspond to both high and low risk alleles^12–14^. Significant increases in Parameter 1 values for at least one pathogenic reporter were identified for 10 of 21 compounds, including: iodoacetamide, PX-12, N-ethylmaleimide, disulfiram, ebselen, carmofur, auranofin, spebrutinib, osimertinib, and necrosulfonamide (Fig. 2A-2B).

Significant increases in Parameter 1 values across half or more of the mutant reporters were identified for 5 of 21 compounds, including: iodoacetamide (13 of 16), N-ethylmaleimide (10 of 16), PX-12 (10 of 16), disulfiram (14 of 16), and auranofin (12 of 16). Of the FDA approved entities, disulfiram and auranofin produced the broadest effects across mutants. Overall, these test compounds had broad effects on pathogenic NOTCH3 variants, though the magnitude of the effects across mutants was heterogeneous.

**Fig. 3:**
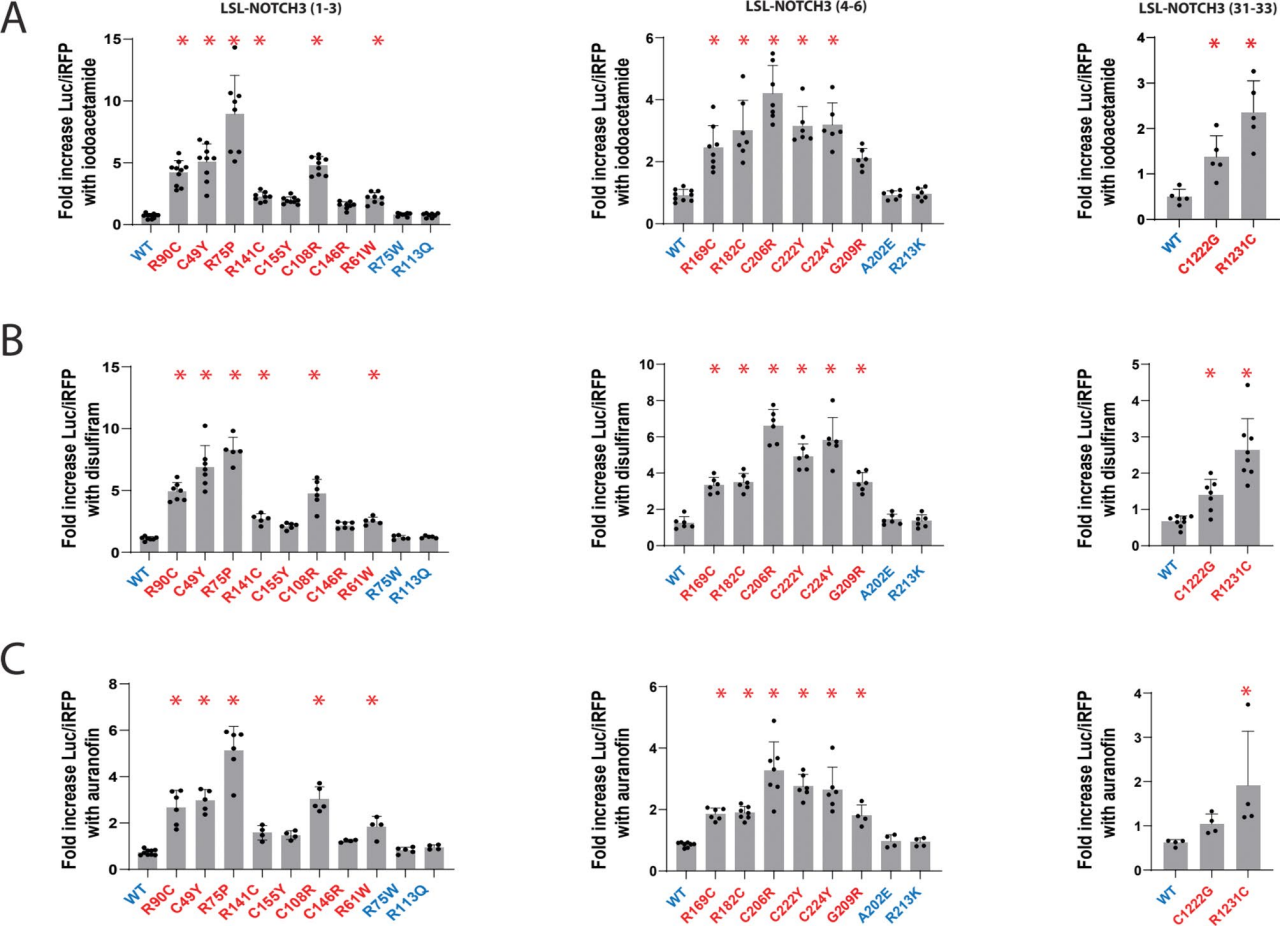
Iodoacetamide, disulfiram, and auranofin as mitigators of pathogenic NOTCH3 conformational alterations. Fold changes in LSL-NOTCH3 activity (Parameter 1; total activity secreted to media) after treatment of transfected cells with iodoacetamide (A; 10 µM), disulfiram (B; 10 µM), and auranofin (C; 1 µM) are shown for reporters that include NOTCH3 EGF repeats 1–3 (left), repeats 4–6 (middle), and repeats 31–33 (right). Wildtype (WT) and benign variants are shown in blue, and pathogenic variants are shown in red. All values are shown with standard deviations. We categorized G209R as pathogenic because it induced gel mobility shifting and substantially suppressed LSL-NOTCH3 activity in a prior study^11^. *p < 0.05 compared to fold increase for WT reporter of each EGF repeat group.

The strongest increases in Parameter 1 values occurred for the following compounds: iodoacetamide (Fig. 3A, up to 12 times relative to wildtype responses), disulfiram (Fig. 3B, up to 7.7 times relative to wildtype responses), and auranofin (Fig. 3C, up to 7.2 times relative to wildtype responses). The primary data for these compounds are presented in Fig. 3. The most potent effects for disulfiram were seen against the R75P and the C206R mutation.

**Fig. 4:**
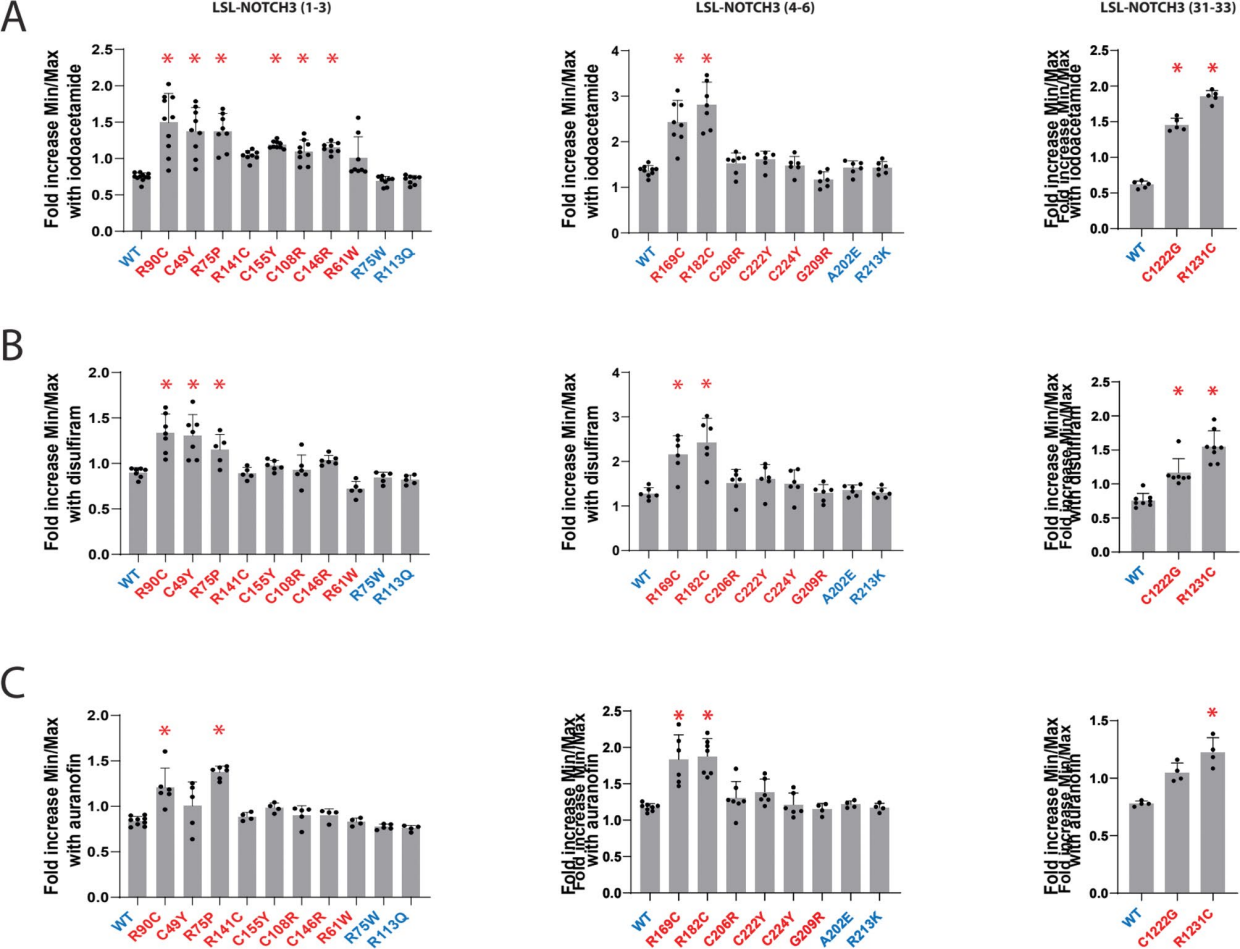
Iodoacetamide, disulfiram, and auranofin increase the fraction of favorable disulfide bonding patterns in NOTCH3 reporters. Fold change in TCEP-independent fraction of LSL-NOTCH3 activity (Parameter 2; min/max after TCEP addition normalized to value control) after treatment of transfected cells with iodoacetamide (A; 10 µM), disulfiram (B; 10 µM), and auranofin (C; 1 µM) are shown for reporters that include NOTCH3 EGF repeats 1–3 (left), repeats 4–6 (middle), and repeats 31–33 (right). Wildtype (WT) and benign variants are shown in blue, and pathogenic variants are shown in red. All values are shown with standard deviations. *p < 0.05 compared to fold increase over WT reporter of each EGF repeat group.

Increases in Parameter 2 values were also identified for 16/21 compounds (Figs. 2C-2D). Thus, a significant majority of compounds elevated both Parameter 1 and 2 for at least one mutant reporter. None of the 21 compounds had any effects on reporters corresponding to non-pathogenic variants of NOTCH3. Figure 4 shows the effects of iodoacetamide (Fig. 4A), disulfiram (Fig. 4B), and auranofin (Fig. 4C) on Parameter 2 across all NOTCH3 variants tested.

### Cell-free versus cell-dependent small molecule effects

**Fig. 5:**
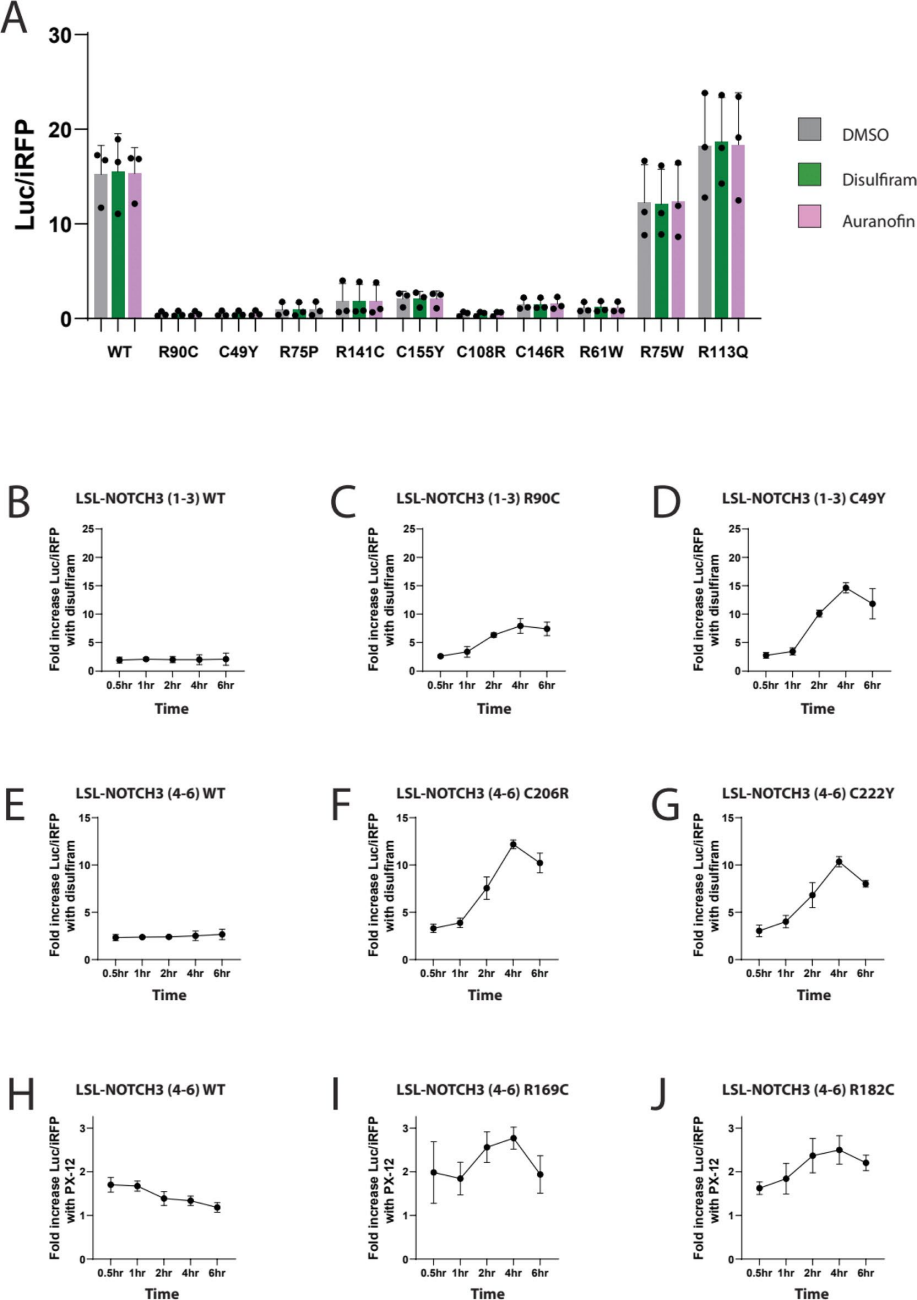
Small molecule mitigation of pathogenic NOTCH3 during cellular processing of target protein. (**A**) To assess cell-independent LSL reporter activity, conditioned media of cells transfected with LSL-NOTCH3 reporters corresponding to the EGF repeat 1–3 variants shown on the x-axis was treated with indicated agents. Luciferase activity was then measured; these were normalized to iRFP expressed in transfected cells (Parameter 1). There were no significant differences that depended on treatment conditions. (**B-J**) To assess if the effects of mitigators of mutant NOTCH3 affected LSL-NOTCH3 reporter activity in a time-dependent fashion, media (without or with mitigators) of cells transfected with WT or mutant reporters indicated were collected over time periods noted in the x-axis. Luciferase activities normalized to without drug control (at the same time point) are displayed on the y-axis. (B-G) were treated with disulfiram (5 µM); (H-J) were treated with PX-12 (5 µM). The NOTCH3 variants (from EGF repeats (1–3) [B-D] and from EGF repeats (4–6) [E-J]) used in reporter constructs are shown. All values are shown with standard deviations.

For compounds that demonstrated activity on CADASIL mutants, we performed experiments on protein after secretion by mixing conditioned media with selected drugs (Fig. 5A). None of these studies showed increases in activity, indicating that the drugs do not act directly on the secreted LSL-NOTCH3 proteins. In time course studies of disulfiram and PX-12 treated cells, there was an increase in Parameter 1 which progressively rose with longer periods of incubation (Fig. 5B-J), which is consistent with an action of these drugs on cellular production and/or processing of the NOTCH3 protein and not on a direct effect on luciferase activity.

### Dose characterization

**Fig. 6:**
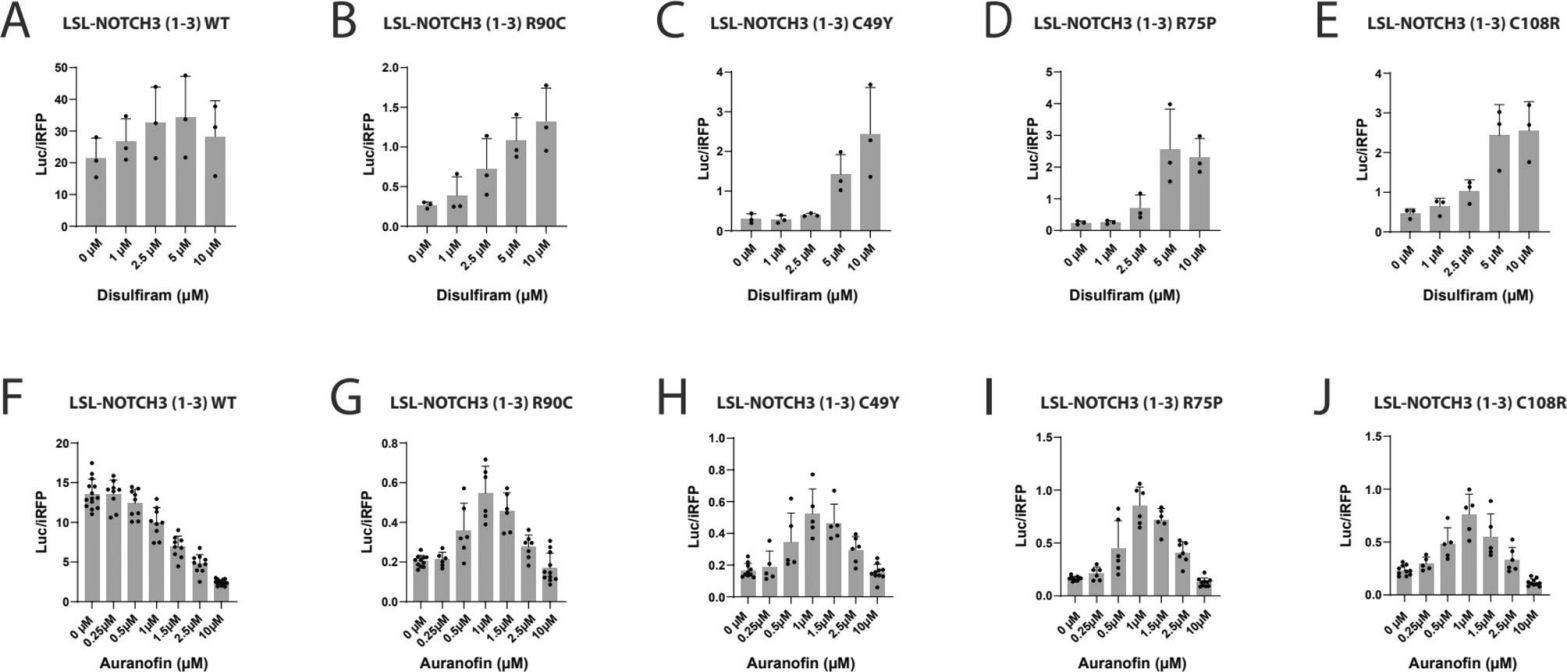
Dose-dependent effects of mitigators on pathogenic NOTCH3 conformational alterations. Media supplemented with doses of disulfiram or auranofin displayed on the x-axis were added to cultures transfected with the indicated LSL-NOTCH3 reporters. The media collected was assayed for luciferase activity which was normalized to iRFP levels and displayed on the y-axis (Parameter 1). All values are shown with standard deviations.

We defined dose/response correlations in a series of studies on disulfiram, PX-12, and auranofin (tested against the most highly responsive mutant reporters; Fig. 6). These targeted studies indicate that the strongest effects on LSL-NOTCH3 production occur with 10 µM disulfiram and 1 µM auranofin.

### Compound effects of cysteine reactive molecules

**Fig. 7:**
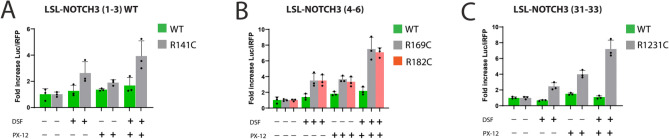
Additive effects of multiple mitigators on pathogenic NOTCH3. For selected LSL-NOTCH3 reporters shown to respond to two different mitigators, we compared the level of luciferase production in media with single drug treatments vs dual treatments (Parameter 1). All drugs were used at 10 µM. NOTCH3 variants were tested from EGF repeats (1–3) (**A**), repeats (4–6) (**B**), and repeats (31–33) (**C**). Values shown are normalized to each reporter’s control expression level without drug treatment. See Supplemental Fig. 1 for reporter expression levels without normalization to no drug controls.

We tested whether combining cysteine reactive drugs resulted in synergistic or additive or antagonistic effects (Fig. 7). Combining PX12 and disulfiram resulted in higher values of LSL-NOTCH3 reporter activity than with each drug used alone; the effects appeared additive.

### Free thiol effects on small molecule potency

**Fig. 8:**
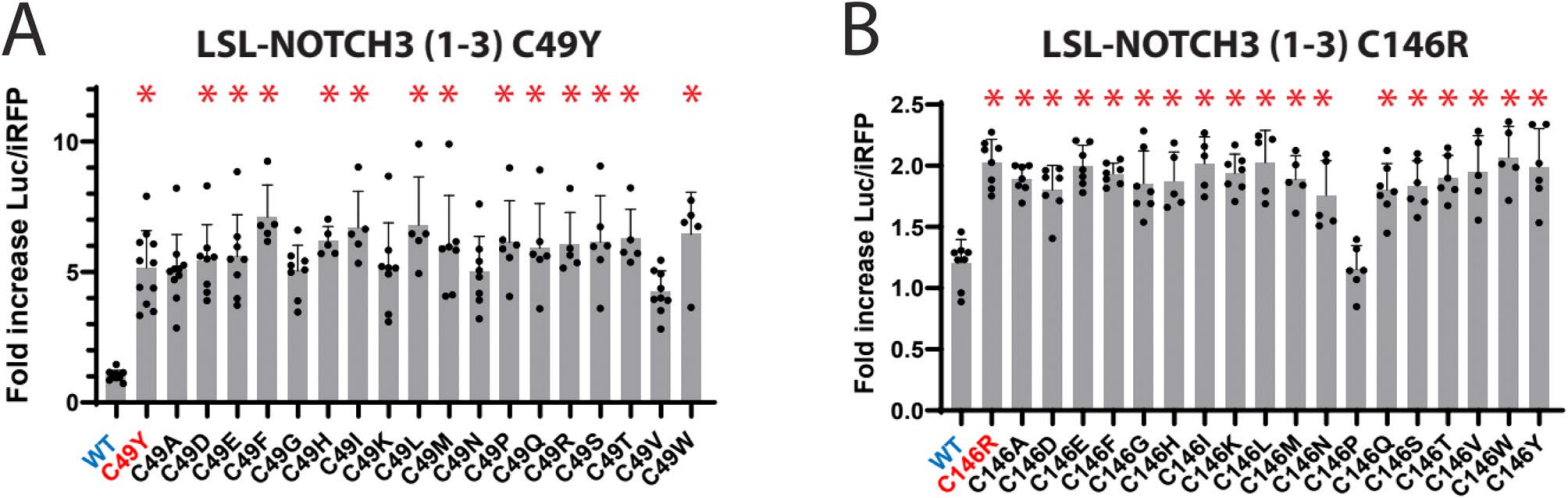
Effect of amino acid residue at cysteine mutation position on mitigator function. A series of mutants which harbor changes at a single cysteine to all other non-cysteine residues were assays for LSL-NOTCH3 activity. In (**A**), NOTCH3 residue 49 of LSL-NOTCH3 (1–3) was mutated from cysteine to all other amino acids. The naturally occurring CADASIL mutation is C49Y. After transfection, cells were treated without or with disulfiram (10 µM), and luciferase in the media quantified. Value of drug induced luciferase activity (Parameter 1) normalized to no drug controls are displayed. (**B**) The same procedure and analysis were used for analysis of NOTCH3 residue 146 of LSL-NOTCH3 (1–3). The naturally occurring CADASIL mutation is C146R. All values are shown with standard deviations. *p < 0.05 compared to fold increase for drug treated WT reporter. See Supplemental Fig. 2 for normalized reporter expression levels for Parameter 2 for this series.

None of the drugs that affected pathogenic NOTCH3 reporters were active on wildtype reporters. Since pathogenic variants are largely cysteine altering mutations that result in a potentially unpaired thiol group, we tested whether drug effectiveness was dependent on the loss of cysteine or, alternatively, on the gain of a non-cysteine amino acid. Accordingly, we mutated the cysteine at residue 49, which was shown to respond well to disulfiram in the C49Y mutant, to all 18 other amino acids (Fig. 8A). Nearly all of the mutants responded to disulfiram, with increased reporter levels. At residue 146, which responds to PX-12 in the pathogenic C146R mutant, alteration to all 18 other amino acids resulted in similar responses to PX-12, except for one mutant (Fig. 8B). These two cases are consistent with the notion that small cysteine reactive compounds act on NOTCH3 because of a loss of cysteine rather than because of a gain of another amino acid.

### Effects of cysteine-reactive drugs on alternative NOTCH3 conformational reporters

**Fig. 9:**
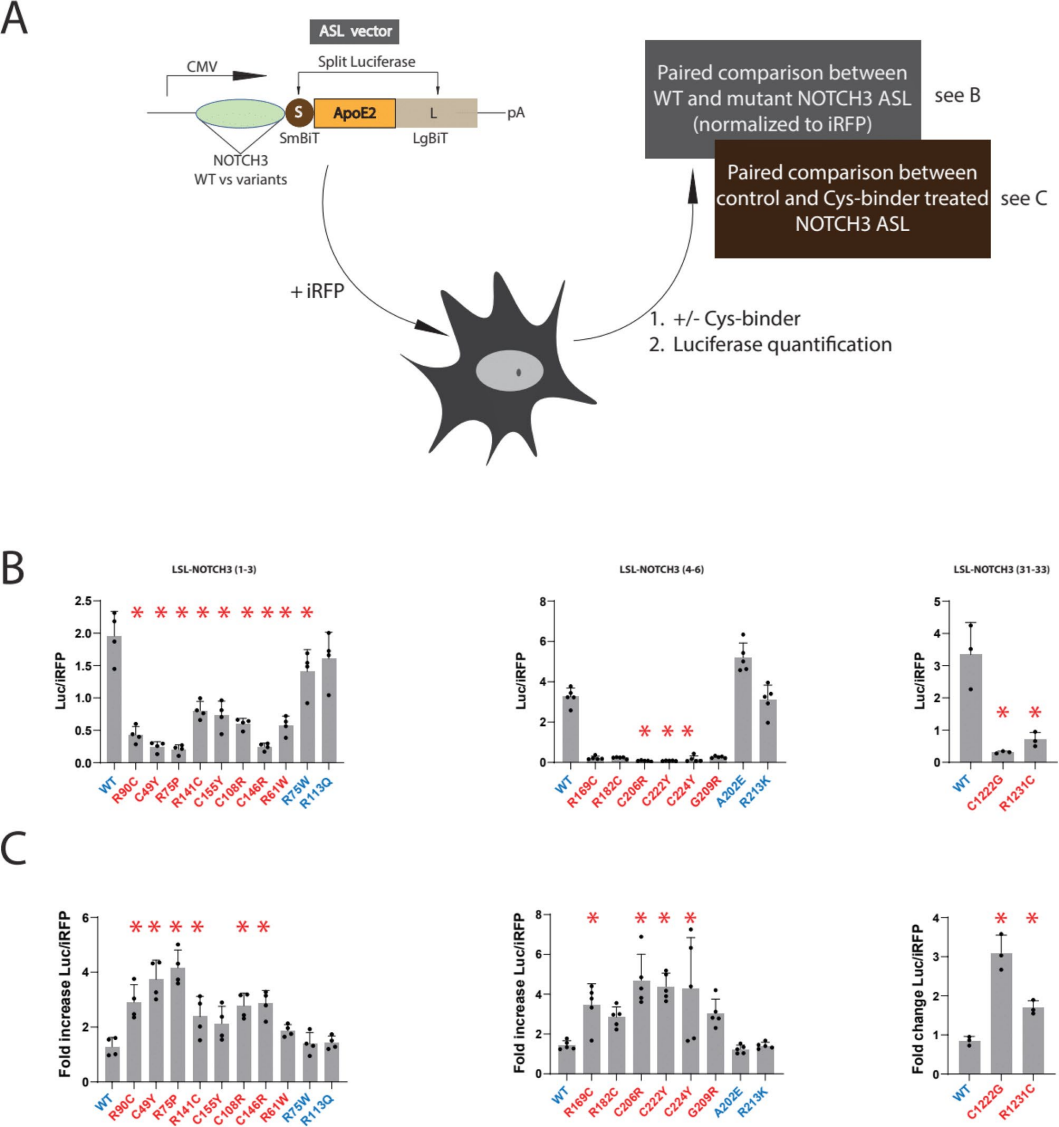
Mitigator effects on an alternative mutant NOTCH3 reporter system. (**A**) Schematic of an APOE-based split luciferase system for differentiating pathogenic NOTCH3 conformations. The system is similar to the LSL-NOTCH3 system except that NOTCH3 sequences of interest are cloned at the 5’ end and inverted split luciferase is flanked by APOE2. Cloned NOTCH3-ASL constructs bearing WT versus variant NOTCH3 fragments are transfected into cells and culture media assayed for activity. To test activity of mitigators of NOTCH3 conformational changes, media is supplemented with candidate small molecules before culture media assays. Transfection efficiency is controlled by measurement of iRFP expression. (**B**) Variant NOTCH3 sequences from EGF repeats (1–3) or (4–6) or (31–33) shown were cloned into the NOTCH3-ASL vector. After transfection into 293 cells, normalized luciferase levels were determined. Conformational alterations in pathogenic mutants (red) were compared to WT and benign variants. Reduction of luciferase activity in pathogenic mutants is consistent with conformational alterations of CADASIL NOTCH3. All values are shown with standard deviations. *p < 0.05 compared to WT reporter. (**C**) NOTCH3-ASL plasmids from (B) were transfected and treated without and with disulfiram (10 µM). Conditioned media from drug treated groups were normalized to media without treatment on the y-axis. All values are shown with standard deviations. *p < 0.05 compared to fold increase for drug treated WT reporter. The left panels of (B) and (C) show results for EGF repeats (1–3), the center panels correspond to EGF repeats (4–6), and the right panels are results for EGF repeats (31–33). WT and benign variants are in blue, and pathogenic variants are in red.

To confirm whether drugs are capable of attenuating conformational changes of pathological NOTCH3 variants under other circumstances, we tested an alternative to the LSL-NOTCH3 assays. This assay, NOTCH3-ASL (APOE split luciferase), is illustrated in Fig. 9A. The test variant of NOTCH3 is cloned to the N-terminus of an inverted split nano-luciferase that is separated by an APOE2 open reading frame with a linker sequence. As shown in Fig. 9B, wildtype and benign NOTCH3 variants generated significantly higher secreted luciferase activity than all pathological mutants, which is consistent with the pathological NOTCH3 generation of abnormal protein that is poorly secreted or synthesized. Cells transfected with a series of pathogenic and non-pathogenic mutants were treated with disulfiram which resulted in increased levels of reporter expression in all pathogenic variants (Fig. 9C). WT NOTCH3-ASL expression was not affected by drug treatment. Because similar drug responses were observed using two reporter systems, it is likely that the drug effects reflect action upon mutant NOTCH3.

### Effects of cysteine-reactive drugs on FBN1-related mutant protein

**Fig. 10:**
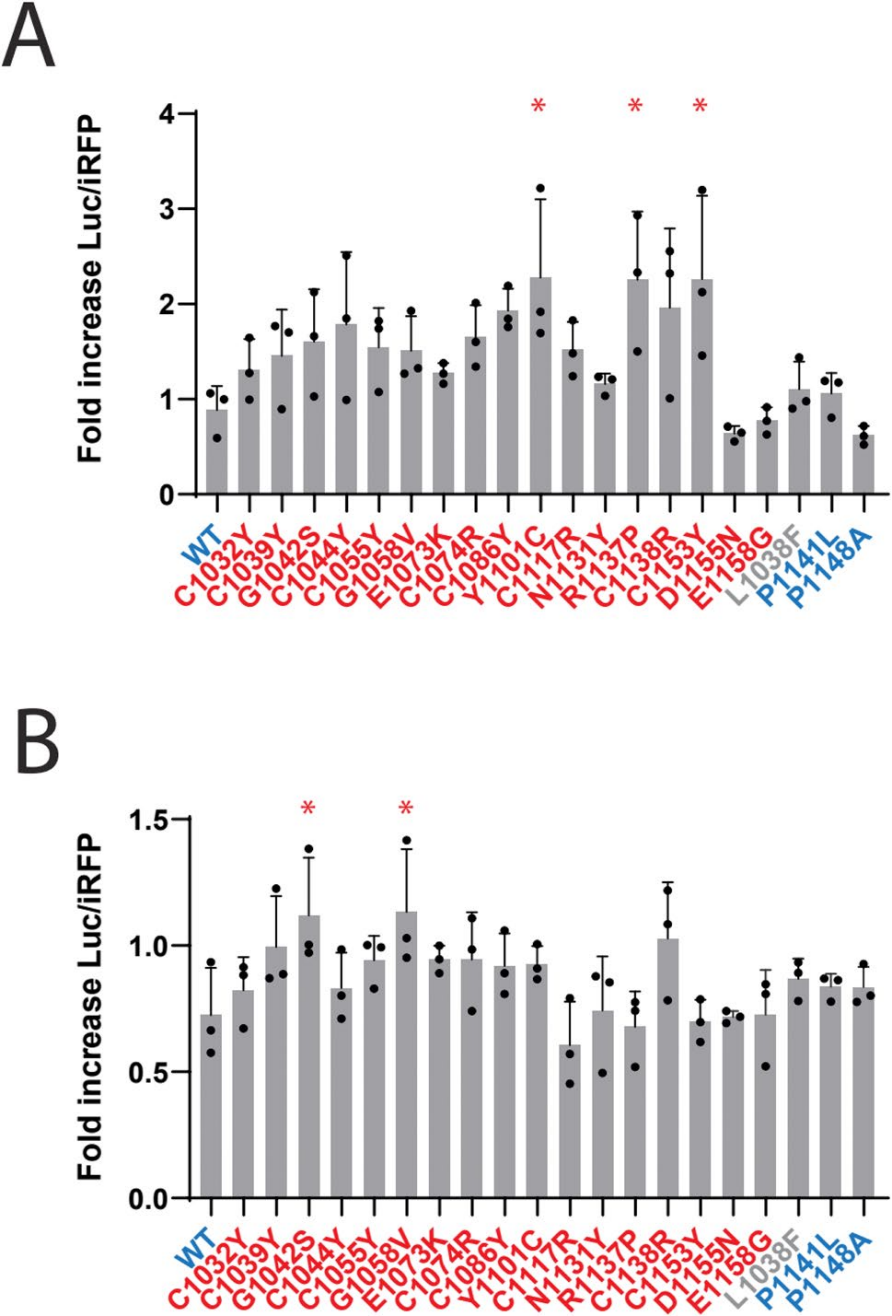
Mitigator effects on FBN1 mutations linked to Marfan syndrome. Previously described LSL-FBN1 reporters^11^ with WT and benign variants (blue) or pathogenic mutations (red) were transfected as in LSL-NOTCH3 experiments. The L1038F variant (gray) is considered a variant of uncertain significance. Media with and without disulfiram (10 µM; (**A**)) or auranofin (1 µM; (**B**)) were assayed for luciferase activity, and values normalized to media without drug for each reporter is shown on the y-axis. All values are shown with standard deviations. *p < 0.05 compared to fold increase for drug treated WT reporter. See Supplemental Fig. 3 for normalized reporter expression levels for Parameter 2 for this series.

Cysteine imbalanced proteins are also implicated in other genetic disorders. In Marfan syndrome, mutations found in the EGF-like repeats cause vascular degenerative changes that are, to date, untreatable^15^. Many of the mutations are similar to those found in CADASIL: they alter cysteine number that results in thiol imbalanced proteins. Accordingly, we tested if LSL-FBN1 reporters that correspond to Marfan mutations could be enhanced by disulfiram or auranofin. Disulfiram increased select pathogenic LSL-FBN1 reporters as shown in Fig. 10A. In contrast, auranofin failed to increase activity of any of the LSL-FBN1 reporters (Fig. 10B). None of the benign mutants in FBN1 displayed beneficial responses to either drug in reporter assays.

## Discussion

Because CADASIL results from mutations that change cysteine number in NOTCH3, thiol imbalance of NOTCH3 and subsequent conformational changes in the protein are suspected to be the initiating steps in disease. This study was motivated by the need to identify approaches that could attenuate pathogenic NOTCH3 conformations. Using a rationally based candidate approach, we hypothesized that small molecules that target thiol residues could selectively attenuate pathogenic NOTCH3 conformations. Our results demonstrate that 1) multiple cysteine-reactive small molecules have the capacity to mitigate the aberrant properties of CADASIL-related NOTCH3 mutants; and 2) select cysteine-reactive small molecules act broadly against subsets of NOTCH3 mutants.

### Cysteine-reactive small molecules act on mutant NOTCH3

This work demonstrates the feasibility of exploiting cysteine reactivity to counter effects of NOTCH3 conformational changes in CADASIL. In view of their shared thiol reactivity, it is likely that the small molecules highlighted here target NOTCH3 via covalent interactions with cysteine residues. One mechanism that is consistent with our results is that mutant NOTCH3 harbors unpaired thiols that react with other thiols of NOTCH3, rendering an aberrant protein secondary structure; however, in the presence of thiol reactive mitigator compounds these unpaired thiols are capped, thereby preventing abnormal intramolecular disulfide bonding. Another possibility is that unpaired thiols of mutant NOTCH3 react with other cellular molecules that bind to cysteine; thiol reactive mitigators may therefore also react with unpaired thiols to prevent pathogenic intermolecular interactions. In support of these mechanisms, we found improvement in Parameter 2 (fraction of properly disulfide bonded protein) in many of the drug/variant combinations (Figs. 3–4) and documented that effects of mitigators on multiple NOTCH3 mutants were independent of non-cysteine residue identity (Fig. 8).

Treatment of protein outside of cells did not change reporter activity, indicating that mitigators act on mutant NOTCH3 inside of cells, potentially when unpaired thiols are initially generated during protein synthesis and maturation. The unavailability of thiols to drugs after synthesis and secretion is consistent with the unexpectedly low amount of thiols in purified mutant NOTCH3 preparations that we previously described for a series of purified NOTCH3 mutants^16^.

### Mitigation of both cysteine and non-cysteine NOTCH3 mutants

We note that non-cysteine mutants (R61W, R75P, and G209R) respond to mitigators, with higher magnitude than several cysteine mutants. Thus, cysteine targeting appears to beneficially affect both cysteine and non-cysteine pathogenic NOTCH3 mutants. In prior work, we note that the non-cysteine mutants have similar redox-dependent gel shift properties as cysteine mutants^10^; furthermore, Parameter 2 non-cysteine and cysteine mutants in LSL-NOTCH3 assays are also uniformly depressed^11^. Thus, experimental evidence points to the possibility that the molecular basis of both cysteine and non-cysteine NOTCH3 mutant impairment may be aberrant disulfide bond formation.

The drug screening used an LSL reporter system, prompting us to test and confirm that mitigation by cysteine-reactive drugs was conserved in an independent genetic reporter system, ASL, that utilizes fused APOE2 with NOTCH3 variant sequences (Fig. 9). We speculate that unreduced cysteine residues of APOE2 in the ASL system may interact with mispaired thiols of pathogenic NOTCH3 variants, enabling discrimination of mutants, but confirmation of this remains to be determined.

The lower degree of benefit across a broad spectrum Marfan syndrome reporters underscores differences between NOTCH3 and FBN1 disease pathophysiology and indirectly supports the role of cysteine imbalance in CADASIL. Whereas NOTCH3 disease is likely a cysteine-imbalance problem, FBN1 disorders such as Marfan syndrome have been proposed to result from independent mechanisms^17,18^. Further, the action of similar drugs on both NOTCH3 and FBN1 emphasizes that the mechanism of action of these FDA-approved drugs is not highly specific.

### Addressing the challenge of molecular heterogeneity of NOTCH3 mutants in CADASIL

One potential challenge in targeting NOTCH3 in CADASIL is molecular heterogeneity, as hundreds of different mutations have been described. In principle, heterogeneity could restrict the utility of highly specific NOTCH3 conformation targeting drugs, which may not work broadly across the patient population. This study suggests an approach to neutralize issues surrounding molecular heterogeneity: using lower specificity cysteine-targeting compounds against mutant NOTCH3.

The broadest NOTCH3 mitigators, disulfiram and auranofin, altered NOTCH3 reporter activity in a majority of mutations across seven EGF repeats and were also effective on gain and loss of cysteine mutations and against non-cysteine pathogenic mutants. Both of these drugs are clinically utilized for alcohol dependency and for rheumatoid arthritis, respectively. The chemical structures of these agents make them unlikely to be highly specific for a narrow range of cysteines^19–25^. Tian et al. recently showed that a multiple cysteine-targeting drugs in fact possess broader than initially realized thiol reactivity ranges^26^. As such, further development of CADASIL targeting drugs could benefit from de-prioritizing complexity and specificity of small molecule drugs and, rather, emphasizing chemical reactivity.

### Limitations and future work

More work is required to determine whether the cysteine-targeted agents identified here also affect the downstream events of CADASIL that have been described. These areas of inquiry will require robust characterization of human cellular models such as differentiated pluripotent stem cells that reflect molecular marker changes that are seen in CADASIL. Further, the studies presented here rely on an in vitro model of NOTCH3 structural changes in protein fragments. Ideally, the agents should also be trialed in animal models of CADASIL expressing full length, mutant NOTCH3, a currently challenging objective^27^. But, on a positive note, many of the agents which show effects on NOTCH3 mutant protein in this study (eg disulfiram and auranofin) are already FDA approved, which may shorten the timeline for clinical testing of efficacy.

In summary, this work validates a discovery strategy that leverages thiol reactivity to impart therapeutic effects on pathological NOTCH3. The application of the LSL assay system study provides a simple and rapid tool to facilitate derivative compound development and for exploration of expanded libraries of cysteine-reactive candidates. The results support the potential for repurposed drugs such as disulfiram and auranofin in NOTCH3 disease which is not restricted to specific mutations. Expansion of the approach provides a foundation for future potential optimization, testing, and deployment of therapeutics against currently untreatable conditions.

## Materials and methods

### Candidate mitigators

Cysteine-reactive small molecules were purchased in purest form available from Sigma-Millipore, MolPort, TOCRIS Bioscience, Med-Chem Express, APExBIO, AdipoGen, and Thermo Fisher Scientific. All reagents were dissolved in DMSO and diluted in the same solvent concentrations that corresponded to 1000-fold of amounts used in cell culture experiments.

### DNA constructs

Expression constructs for conducting the LSL assay have been previously described^11^, and new constructs for the current study were built on the same platform DNA vectors. Point mutations in NOTCH3 were incorporated by PCR of templates from mutants described^10^ or by nested PCR using oligos that included desired mutations. DNA fragments were digested with restriction enzymes and cloned into vectors with T4 DNA ligase. Clones were sequenced prior to use in expression assays. Point mutations of FBN1 have been previously described^11^. For NOTCH3-ASL constructs in Fig. 9, NOTCH3 EGF repeats 1–3 replaced the light chain sequence of the LSL vector by PCR; a secretion peptide sequence at the 5’ end was incorporated into all clones. Subsequently, the full length human APOE2 open reading frame (with its stop codon deleted, incorporating flanking SalI/AgeI and SphI/BglII sites at the 5’ and 3’ ends of the ORF) replaced the NOTCH3 sequences of LSL clones to produce the reporter system shown in Fig. 9A.

### Cell culture and small molecule treatments

HEK293 cells (293A; Qbiogene) were grown in DMEM with 10% fetal bovine serum in 5% carbon dioxide chambers. Gene transfection was conducted with PolyJet as recommended by the manufacturer. As before^11^, 400 ng of LSL vector and 100 ng of iRFP plasmid were mixed in 25µL DMEM. DMEM (25µL) and 1.5µL PolyJet were added to diluted DNA and then dropped onto media of cells in 24 well plates. After 18–24 h, the media was exchanged with OptiMEM supplemented with 0.1% DMSO or test drug at the concentrations described; stocks of drug were in DMSO and diluted 1:1000 into OptiMEM. After 2 h, conditioned media was removed for analysis of nanoLuc activity.

### Split luciferase analysis of small molecule activity on pathogenic mutants

LSL-NOTCH3 experiments were performed according to protocols described in prior work^11^, with all experiments on mutant sequences compared to appropriate wildtype NOTCH3 sequence cloned into the LSL vector. The effects of small molecules were determined by comparing two luciferase parameters with and without candidate mitigators of interest (Fig. 1). Parameter 1 (secreted nanoLuc activity normalized to iRFP) was determined as before^11^ (25µL conditioned media combined with 6.25 µL reaction mixture). Luciferase activity was determined in which plastic plates in a plate reading luminometer (BioTek Synergy LX multi-mode reader). In all experiments, unless noted, the candidate mitigator response was Luc/iRFP with small molecule referenced to without. For determination of Parameter 2 (reduction-unmasked Luc activity), 2 µL of TCEP (31.25 mM) was mixed with the reaction mixture and luciferase activity was followed over 30 min. Time course values were fitted to the equation: Y = Y0 + (Plateau-Y0)*(1-exp(-K*x)) to derive the plateau (max) and the initial value before adding TCEP was deemed the Min level. The Min/Max ratio was determined, corresponding most likely to the percentage of protein in normal conformation relative to total protein. Unless noted, the Min/Max with candidate mitigators was normalized to samples without candidate exposure. Parameter 3 from^11^ was not determined in this study. The LSL-NOTCH3 protocol was used to analyze NOTCH3-ASL reporters from Fig. 9, except that TCEP challenges were not performed (only Parameter 1 was assessed). The LSL-NOTCH3 methods described above were used for FBN1 analysis in Fig. 10.

### Statistics

Normality was assessed by the Shapiro–Wilk test. Significant differences were determined using one-way ANOVA with Dunn’s multiple comparisons test using GraphPad Prism 10.4.1. A *p* value < 0.05 was considered statistically significant.

## Supplementary Information

Supplementary Information.

## Data Availability

All data generated or analyzed during this study are included in this published article [and its supplementary information files].
