# Supplementary material for: Cysteine-reactive mitigators of small vessel disease-related NOTCH3 mutants
Source: Sci Rep. 2026 Mar 20;16:14300. doi: 10.1038/s41598-026-45103-1 (PMC13144312; doi:10.1038/s41598-026-45103-1)

SUPPLEMENTARY DATA

Supplemental Figure 1. Effect of dual treatments on LSL-NOTCH3 reporters. This is a companion to Figure 7, which shows normalized Parameter 1 values for treatments with disulfiram (DSF) and PX-12. (A-C) shows values of reporter activity for experiments without normalization to WT reporter responses to drugs and corresponds to Fig 7A-7C. (D-F) shows unnormalized Parameter 2 values for the same experiments. (G-I) shows normalized Parameter 2 values for the same experiments, where the mitigator-induced changes in Parameter 2 of mutant reporters were referenced to those of the WT reporter. Although mitigators had relative effects on pathogenic mutations, the values did not rise to wildtype levels. For example, disulfiram increased the absolute value of Parameter 1 for the R141C mutant from 7.9% to 19.4% of the wildtype without disulfiram value (2.5-fold change with drug). Meanwhile, PX-12 increased Parameter 1 for the R182C mutant from 6.7% to 21.1% of the wildtype without drug value (3.1-fold change with drug).

Supplemental Figure 2. Changes in disulfide conformation normalization in mutants at the C49 and C146 positions of NOTCH3. This is a companion to Figure 8 and shows the Parameter 2 values for Fig 8A-8B. All Parameter 2 values of mutant reporters were referenced to those of the WT reporter. Changes indicate that candidates are capable of favorably changing the fraction of NOTCH3 to non-pathological configurations. All values are shown with standard deviations. * $p<0.05$ compared to fold increase for drug treated WT reporter.

Supplemental Figure 3. Changes in disulfide conformation normalization in FBN1 mutants. This is a companion to Figure 10 and shows the Parameter 2 values for Fig 10A-10B. All Parameter 2 values of mutant reporters were referenced to those of the WT reporter. Drugs that were assessed were disulfiram (10µM; (A)) and auranofin (1µM; (B)). Changes indicate that candidates are capable of favorably changing the fraction of NOTCH3 to non-pathological configurations. All values are shown with standard deviations. * $p<0.05$ compared to fold increase for drug treated WT reporter.

Supplemental Figure 1

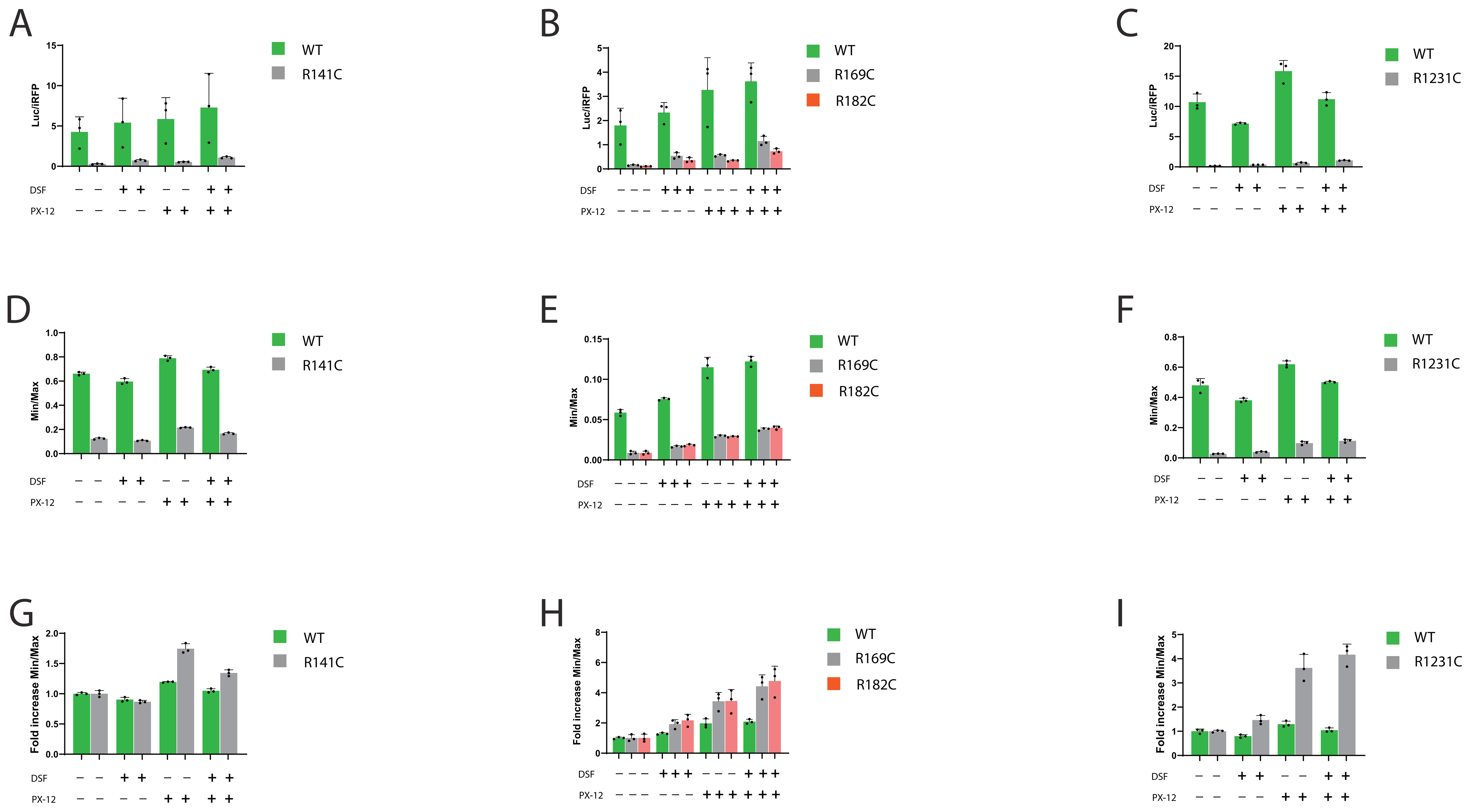

Supplemental Figure 2

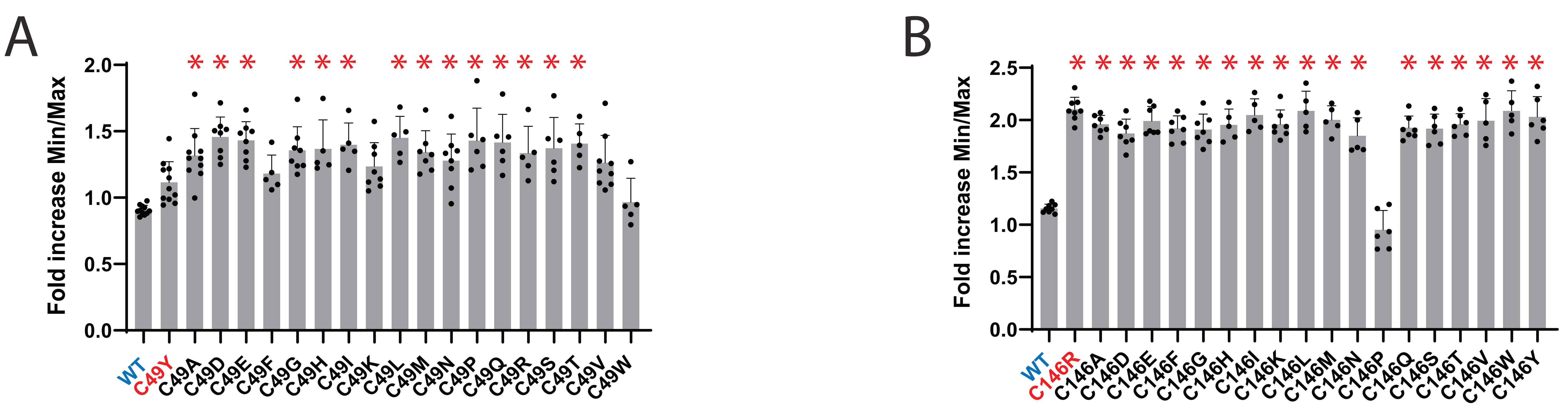

Supplemental Figure 3

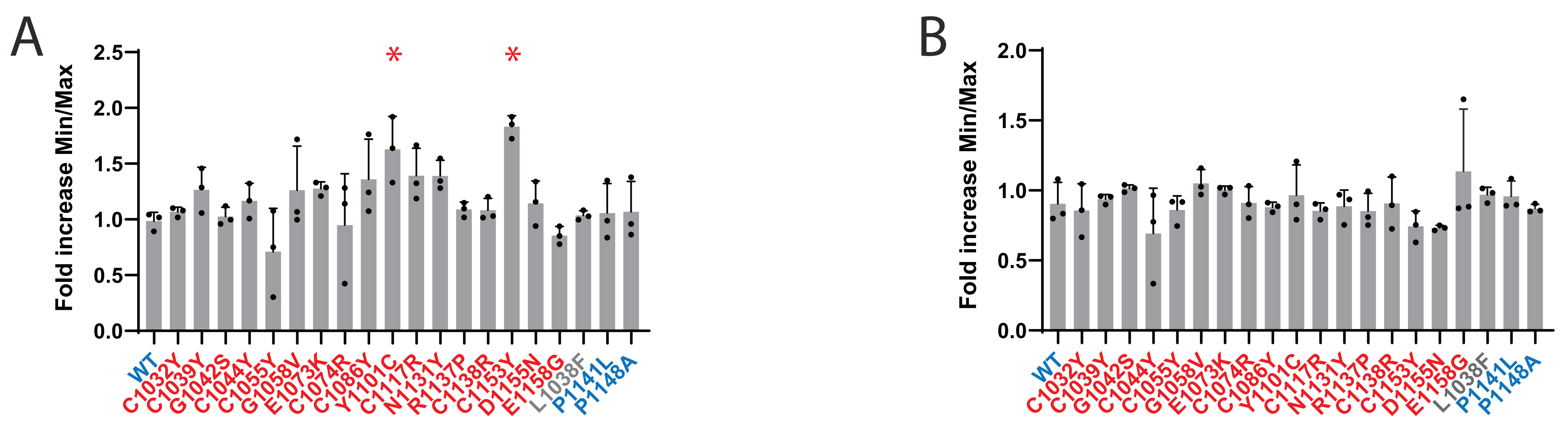

Supplement: Supplementary file 1 — Supplementary Information. [file 41598_2026_45103_MOESM1_ESM.pdf]
